# Standards of care for hypoparathyroidism in adults: a Canadian and International Consensus

**DOI:** 10.1530/EJE-18-0609

**Published:** 2018-12-10

**Authors:** Aliya A Khan, Christian A Koch, Stan Van Uum, Jean Patrice Baillargeon, Jens Bollerslev, Maria Luisa Brandi, Claudio Marcocci, Lars Rejnmark, Rene Rizzoli, M Zakarea Shrayyef, Rajesh Thakker, Bulent O Yildiz, Bart Clarke

**Affiliations:** 1 McMaster University, Hamilton, Ontario, Canada; 2 Technische Universitat Dresden, Dresden, Germany; 3 Western University, London, Ontario, Canada; 4 Université de Sherbrooke, Sherbrooke, Quebec, Canada; 5 Oslo University Hospital, Oslo, Norway; 6 University of Florence, Florence, Italy; 7 University of Pisa, Pisa, Italy; 8 Aarhus University, Aarhus C, Denmark; 9 University Hospital of Geneva, Geneva, Switzerland; 10 University of Toronto, Toronto, Canada; 11 University of Oxford, Oxford, UK; 12 Hacettepe University School of Medicine, Ankara, Turkey; 13 Mayo Clinic, Rochester, Minnesota, USA

## Abstract

*Purpose:* To provide practice recommendations for the diagnosis and management of hypoparathyroidism in adults.

*Methods:* Key questions pertaining to the diagnosis and management of hypoparathyroidism were addressed following a literature review. We searched PubMed, MEDLINE, EMBASE and Cochrane databases from January 2000 to March 2018 using keywords ‘hypoparathyroidism, diagnosis, treatment, calcium, PTH, calcidiol, calcitriol, hydrochlorothiazide and pregnancy’. Only English language papers involving humans were included. We excluded letters, reviews and editorials. The quality of evidence was evaluated based on the Grading of Recommendations Assessment, Development and Evaluation (GRADE) approach. These standards of care for hypoparathyroidism have been endorsed by the Canadian Society of Endocrinology and Metabolism.

*Results:* Hypoparathyroidism is a rare disease characterized by hypocalcemia, hyperphosphatemia and a low or inappropriately normal serum parathyroid hormone level (PTH). The majority of cases are post-surgical (75%) with nonsurgical causes accounting for the remaining 25% of cases. A careful review is required to determine the etiology of the hypoparathyroidism in individuals with nonsurgical disease. Hypoparathyroidism is associated with significant morbidity and poor quality of life. Treatment requires close monitoring as well as patient education. Conventional therapy with calcium supplements and active vitamin D analogs is effective in improving serum calcium as well as in controlling the symptoms of hypocalcemia. PTH replacement is of value in lowering the doses of calcium and active vitamin D analogs required and may be of value in lowering long-term complications of hypoparathyroidism. This manuscript addresses acute and chronic management of hypoparathyroidism in adults.

*Main conclusions:* Hypoparathyroidism requires careful evaluation and pharmacologic intervention in order to improve serum calcium and control the symptoms of hypocalcemia. Frequent laboratory monitoring of the biochemical profile and patient education is essential to achieving optimal control of serum calcium.

## Introduction

Hypoparathyroidism is an uncommon disorder that is characterized by hypocalcemia and hyperphosphatemia due to low or inappropriately normal serum levels of parathyroid hormone (PTH). The most common cause of hypoparathyroidism is neck surgery, (75% of the cases) during which parathyroid glands are inadvertently injured, removed or deprived of their blood supply. The causes of nonsurgical hypoparathyroidism which comprise the remaining 25% of the cases are listed in Table 1. PTH resistance syndromes have the same biochemical profile as hypoparathyroidism however PTH levels are elevated.

**Table 1:** Causes of nonsurgical hypoparathyroidism.

•Autoimmune •Genetic variantsInfiltrative •MetastaticRadiation destruction •Mineral deposition (copper or iron) •Functional (magnesium deficiency or excess) •Transient (burn injuries, acute illness) •PTH resistance states •Maternal hyperparathyroidism •Idiopathic

Conventional therapy of hypoparathyroidism consists of the use of calcium supplements, and active vitamin D. This therapeutic approach addresses the hypocalcemia of hypoparathyroidism, but fails to provide a physiologic replacement for the lack of PTH. Replacement therapy with recombinant human PTH (rhPTH(1–84)) is now approved in the United States and Europe as an adjunctive treatment for adult patients with chronic hypoparathyroidism who cannot be well controlled on conventional therapy management of hypoparathyroidism during pregnancy is challenging with very limited data currently available to guide clinical practice. Canadian Endocrine Update, McMaster University, Western University and the Mayo Clinic formed a working group comprising of parathyroid experts as well as general endocrinologists. Key questions addressing the diagnosis and management of hypoparathyroidism were addressed following an extensive review of the literature. These recommendations reflect current evidence and consensus regarding the appropriate standard of care today. These practice recommendations are to be applied in the context of clinical care with appropriate adjustments for comorbidities, individual preferences as well as other patient factors. They do not preclude clinical judgment and reflect the limited clinical trial evidence available today.

## Methodology

**Figure 1 fig1:**
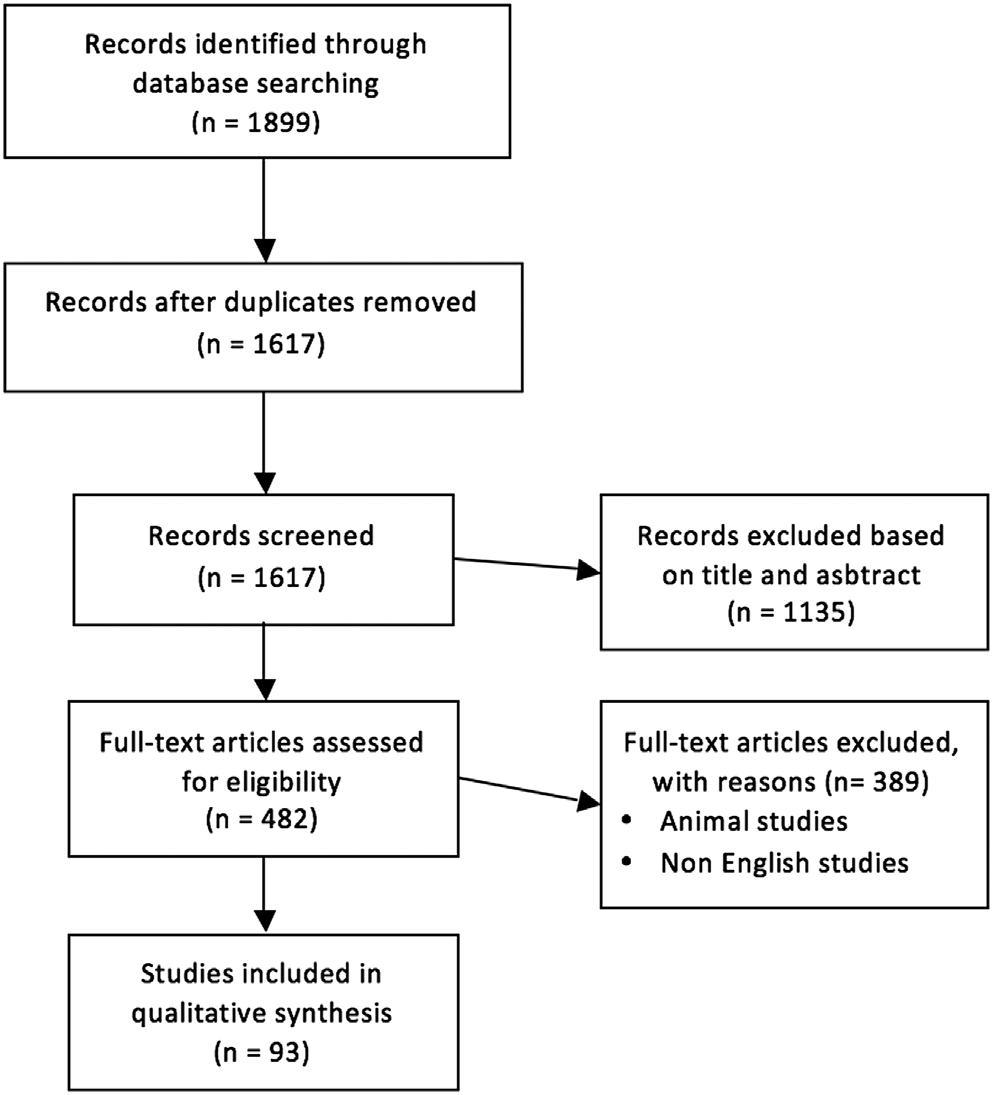
Flow diagram.

We searched PubMed, MEDLINE, EMBASE and Cochrane databases from January 2000 to March 2018 using keywords ‘hypoparathyroidism, diagnosis, treatment, calcium, PTH, calcidiol, calcitriol, hydrochlorothiazide and pregnancy’. MeSH terms were utilized in various combinations to increase search sensitivity. The search strategy was adapted to PubMed to search for articles published ahead of print (Figure 1). Of the 482 citations, we included only English language papers involving humans. We excluded letters, reviews and editorials. As evidence is limited for certain questions being addressed, we included case reports and case series. Evaluation of the quality of evidence was performed based on the Grading of Recommendations Assessment, Development and Evaluation (GRADE) approach (1) (Table 2). Papers were identified by content experts (A K and B C). Landmark papers published earlier then 2000 were also included if the findings have not been superseded by more recent data. All co-authors contributed to the development of this standards paper. The key questions, results of the literature review and practice recommendations were presented at the Endocrine Society annual meeting in March 2018 in a symposium session entitled ‘2018 Parathyroid Summit: A Focus on Hypoparathyroidism.’ The session included time for attendee feedback, which was incorporated into the current manuscript.

**Table 2:** Quality assessment criteria (1).

Study design	Quality of evidence	Lower if	Higher if
Randomized trial	High	Risk of bias	Large effect
		(−1) Serious	(+1) Large
		(−2) Very serious	(+2) Very large
	Moderate	Inconsistency	Dose response
		(−1) Serious	(+1) Evidence of a gradient
		(−2) Very serious	
Observational study	Low	Indirectness	All plausible confounding
		(−1) Serious	(+1) Would reduce a demonstrated effect or
		(−2) Very serious	
	Very low	Imprecision	(+1) Would suggest a spurious effect when results show no effect
		(−1) Serious	
		(−2) Very serious	
		Publication bias	
		(−1) Likely	
		(−2) Very likely	

This concise document outlines acceptable care today based on current evidence and consensus. We provide tables and tools to simplify, enhance and standardize treatment.[Fig fig1]

## 1. Confirming a diagnosis of hypoparathyroidism and evaluating target organ damage

(a) How is chronic hypoparathyroidism diagnosed?

In patients with history of thyroid, parathyroid, laryngeal or other neck surgeries, the temporal proximity of the surgical procedure to the development of hypocalcemia, as well as the duration of hypocalcemia, will determine whether this is acute and/or transient hypoparathyroidism (surgery <6 months prior) or chronic and permanent hypoparathyroidism (surgery >6 months prior) (2, 3, 4, 5, 6, 7). A low serum albumin-adjusted calcium <2.0 mmol/L or <8 mg/dL in the presence of a low PTH <15 pg/mL is associated with a high risk of permanent hypoparathyroidism and requires appropriate supplementation with calcium and active vitamin D metabolites and follow-up (8, 9).

Hypocalcemia for six or more months after surgery, with or without symptoms in the presence of a low or inappropriately normal PTH is diagnostic of permanent surgical hypoparathyroidism. Classic symptoms of hypoparathyroidism principally reflect the effects of hypocalcemia, and may involve neuromuscular, neurologic, behavioral/psychiatric and cardiovascular systems and include numbness and tingling in face, hands or feet, muscle cramping, twitching, depression, irritability, confusion, seizures, bradyarrythmias, wheezing and laryngospasm. Laboratory testing should confirm a low albumin-corrected total serum calcium level or a low ionized calcium levels and low PTH on at least two occasions (emergent care for hypocalcemia should not be delayed and does not require two test results for confirmation of hypocalcemia). If the PTH is in the normal reference range and is not elevated in the presence of hypocalcemia, this would also be indicative of impaired parathyroid response to the hypocalcemic insult and diagnostic of hypoparathyroidism (3). In nonsurgical patients, obtaining the family history is essential along with assessing other clinical features that are part of complex syndromes in which hypoparathyroidism is a component (4, 5). Genetic testing is available to diagnose the molecular basis for the disorder (Table 3).

**Table 3:** Genetic causes of hypoparathyroidism – key clinical findings and lab tests.

Disorder	Clinical or laboratory features prompting consideration of specific genetic or other types of testing	Molecular defect	Genetic and other testing to establish diagnosis
Autosomal dominant hypoparathyroidism (ADH) type 1 and 2	Typically asymptomatic or mild hypocalcemia with or without hypercalciuria (ADH types 1 and 2)	Gain of function mutation in CASR (type 1) or G alpha 11 (type 2)	CASR or GNA11 sequencing
ADH type 1 with Bartter’s syndrome type 5	Hypocalcemia, hypomagnesemia, hypokalemia, alkalosis, hypercalciuria, and salt and water depletion may be seen depending on the severity	CASR	CASR sequencing
Isolated hypoparathyroidism	Presentation dominated by biochemical and clinical features of hypoparathyroidism		PTH, GCM2, sequencing depending on presentation
Autosomal recessive		PTH or GCM2	
Autosomal dominant		PTH or GCM2	
X-linked recessive		SOX3 locus (in males)	
Hypoparathyroidism of autoimmune etiology			
Autoimmune mediated			
Autoimmune polyendocrine syndrome type 1 (APS1)Isolated	Other autoimmune diseases and features such as mucocutaneous candidiasis, adrenal insufficiency and hypogonadismMay show only hypoparathyroidism	Usually due to homozygous mutations in AIREAIRE mutations or of unknown etiology	AIRE sequencingPresence of 21-hydroxylase antibodies supports diagnosis of autoimmune adrenal insufficiencyTesting for other hormonal insufficiency states (e.g., adrenal and gonadal insufficiency)AIRE sequencing
Hypoparathyroidism, deafness, renal anomalies (HDR) syndrome	Sensorineural deafness, renal anatomic abnormalities and renal dysfunction, autosomal dominant inheritance	GATA3	GATA3 sequencing, hearing testing, renal imaging
DiGeorge syndrome	Cardiac defects (present in ~80% including ventriculoseptal defect, tetralogy of Fallot, interrupted aortic arch, truncus arteriosus), immunodeficiency (recurrent infections, thymic hypoplasia or aplasia, T cell lymphopenia), hypoparathyroidism, pharyngeal and laryngeal abnormalities, cleft palate, behavioral and psychiatric problems, ophthalmic anomalies, hearing loss	Variety of defects and deletions and microdeletions in chromosome 22q11.2	Fluorescence in situ hybridization (FISH) is the traditional test most commonly doneTwo other diagnostic approaches are used with greater frequency than FISH including PCR-based multiplex ligation-dependent probe amplification and SNP) array. In some case, TBX sequencing is done
Kenny–Caffey syndrome			
Type 1 or Sanjad–Sakati syndrome (autosomal recessive)	Short stature, growth retardation, small hands and feet, cortical thickening and medullary stenosis of the long bones, delayed fontanelle closure, abnormal eyes, dysmorphic facies, hypoparathyroidism	TBCE	TBCE sequencing
Type 2 (autosomal dominant)	Gracile bone dysplasia, short stature with cortical thickening and medullary stenosis of tubular bones, delayed closure of anterior fontanelle, eye abnormalities, and hypoparathyroidism	FAM111A	FAM111A sequencing
Hypoparathyroidism associated with mitochondrial disorders		Mutations in the mitochondrial genome	Mitochondrial DNA sequencing
Kearns Sayre syndrome	Ophthalmoplegia, retinal pigmentary and cardiac conduction abnormalities, proximal and bulbar weakness, possibly ataxia	mtDNA large-scale deletion	Specialized clinical assessments depending on the manifestations (cardiac, ophthalmologic, neurologic, endocrinologic and others)
MELAS	Encephalomyopathy, lactic acidosis, and stroke-like episodes along with external ophthalmoplegia, diabetes, hearing loss, early-onset stroke symptoms, migraine, and cognitive dysfunction	Mutations in the mitochondrial tRNA Leu gene	
MTPDS	Disordered fatty acid oxidation associated with neuropathy, retinopathy and fatty liver	Mutations in mitochondrial genome	

AIRE, autoimmune regulator of endocrine function; CASR, calcium-sensing receptor; GNA11; G protein alpha subunit 11; MELAS, mitochondrial encephalomyopathy, lactic acidosis, and stroke-like syndrome; MTPDS, mitochondrial trifunctional protein deficiency.

## Nonsurgical causes of hypoparathyroidism

### 1. Autoimmune hypoparathyroidism

Autoimmune hypoparathyroidism is the most common cause of nonsurgical hypoparathyroidism. It may occur in isolation or as part of the autoimmune polyendocrine syndrome1 (APS1). APS1 is caused by mutations in the autoimmune regulator gene (*AIRE*), which is expressed in the lymph nodes, thymus, pancreas, adrenal cortex and fetal liver (10, 11).

APS1 is associated with circulating autoantibodies and infiltration of the involved organs with lymphocytes resulting in organ failure. APS1 is characterized by three major clinical features: chronic mucocutaneous candidiasis, hypoparathyroidism and adrenal insufficiency. More than 80% of APS1 patients have hypoparathyroidism, and this may be the only endocrinopathy present (12, 13).

Patients may have in addition more than 20 minor clinical features (3, 5) (Table 4). The diagnosis of APS1 is probable in the presence of at least one major syndrome and positive antibodies to type 1 interferons, which are present in >95% of patients (12). Autoantibodies to 21-hydroxylase correlate with the development of adrenal insufficiency and antibodies to NALP5 correlate with the development of hypoparathyroidism and measuring these antibodies can be of value in predicting the development of adrenal insufficiency and hypoparathyroidism respectively A molecular diagnosis can be confirmed with DNA studies of the *AIRE* gene (14).

**Table 4:** Features of APS1.

**Major features**
•Mucocutaneous candidiasisHypoparathyroidism •Adrenal insufficiency
**Minor features**
•Keratitis •Autoimmune hepatitis •Primary ovarian failure •Enamel hypoplasia •Enteropathy with chronic diarrhea or constipation •Photophobia •Periodic fever with rash •Pneumonitis •Nephritis •Pancreatitis •T1DM •Functional asplenia •Celiac disease •Thyroiditis •Retinitis •Pure red-cell aplasia •Polyarthritis

Activating antibodies to the calcium-sensing receptor can suppress PTH secretion leading to hypoparathyroidism (15). Antibodies against the extracellular domain of the CASR have been identified in individuals with autoimmune hypoparathyroidism (16).

### 2. Genetic variants

Genetic causes of hypoparathyroidism is presented in Table 3.

#### A. Isolated hypoparathyroidism

a. Glial cells missing (GCM2) gene mutation results in isolated hypoparathyroidism with early onset of hypoparathyroidism due to parathyroid gland agenesis (17).

b. Autosomal dominant hypocalcemia (ADH).

Type 1 is caused by an activating mutation in the calcium-sensing receptor (*CASR*) gene which increases the sensitivity of the CASR to extracellular ionized calcium. The mutated receptor is expressed in the parathyroid glands causing suppression of PTH synthesis and secretion at normal ionized calcium levels leading to hypoparathyroidism (17). In the kidney, the mutated receptor results in enhanced urine calcium losses with relative hypercalciuria and an increased risk of nephrocalcinosis and nephrolithiasis.

Type 2 is caused by an activating mutation in the guanine nucleotide-binding protein alpha 11 (*GNA11*) gene encoding the alpha subunit of the G protein G11 – a key mediator of CASR signaling resulting in suppresion of PTH even at low serum calcium levels (18,19). Urine calcium excretion is not affected in type 2 ADH (20).

c. PTH gene mutations:

Rare mutations in the *PTH* gene located on locus 11p15 have been associated with defective synthesis and secretion of PTH (21).

#### B. Hypoparathyroidism with additional features (Table 3)

a. DiGeorge syndrome is a rare condition affecting 1/4000–5000 live births, 70–80% carry a microdeletion within 22q.21–q11.23 chromosome. The clinical features include developmental delay, palatal anomalies, parathyroid aplasia or hypoplasia causing neonatal hypocalcemia, aplastic or hyoplastic thymus with immune deficits, congenital heart defects, facial anomalies, speech and learning disabilities (22, 23, 24).

b. Hypoparathyroidism, sensorineural deafness and renal dysplasia syndrome (HDR syndrome).

This is a rare autosomal dominant disorder associated with mutations or deletions in the GATA-binding protein 3 gene localized to chromosomal region 10p14–15 resulting in haploinsufficiency of the transcription factor GATA 3, a protein critical for normal parathyroid, kidney and otic vesicle development. The presentation includes the presence of hypocalcemia, hearing loss and renal disease (25, 26).

c. Hypoparathyroidism–retardation–dysmorphism (HRD) syndrome. HRD syndrome is a rare form of autosomal recessive hypoparathyroidism which encompasses the two syndromes namely the Sanjad–Sakati and the Kenney Caffey syndromes type 1 and type 2. The clinical features include congenital hypoparathyroidism, severe growth impairment, mental retardation and facial dimorphism (27, 28, 29).

### 3. Infiltrative causes

Destruction of the parathyroid glands secondary to granulomatous infiltration (e.g. sarcoidosis, amyloidosis, Riedel’s thyroiditis) has been reported (30, 31, 32, 33, 34).

### 4. Metastatic cancer

Infiltrating secondary tumors very rarely cause hypoparathyroidism. The most common tumors are breast, leukemia, skin, lung, sarcomas and lymphomas (35).

### 5. Radiation destruction

Rarely high doses of ionizing radiation exposure have been associated with hypoparathyroidism (36, 37, 38).

### 6. Mineral deposition

Wilson’s disease with copper deposition in the parathyroid glands has been reported to be a cause of hypoparathyroidism (39, 40, 41). Hemochromatosis is caused by iron overload and can cause hypoparathyroidism. Iron overload can also occur with repeated transfusions and can develop in individuals with thalassemia (42, 43, 44).

### 7. Transient hypoparathyroidism

Severe burn injuries and acute illness can be associated with transient hypoparathyroidism (45, 46).

### 8. Functional hypoparathyroidism (magnesium deficiency or excess)

Both hypomagnesemia and hypermagnesemia can impair parathyroid function. Abnormalities in serum magnesium require further evaluation and correction (Tables 5 and 6). Magnesium is reabsorbed in the kidney and absorbed in the bowel in a passive paracellular manner (47, 48). Transcellular absorption of Mg^+2^ occurs via the transient receptor potential melastatin subtype 6 (TRPM6) channels in the bowel and kidney (48). Mg^+2^ can activate the CASR and lower PTH synthesis and secretion (49, 50). Activation of the CASR by Mg^+2^ stimulates phospholipase C and A2 and inhibits cellular cAMP with inhibition of PTH release (51). Thus, high serum magnesium levels can decrease PTH and contribute to hypocalcemia. Activation of the CASR in the kidneys results in decreased paracellular transport of both calcium and magnesium in the kidneys.

Activating mutations of the CASR result in ADH with hypocalcemia as well as hypomagnesemia in a significant number of patients (52).

Mutations in the claudin proteins (claudin 16 and 19) impair paracellular renal reabsorption of both calcium and magnesium causing familial hypomagnesemia with hypercalciuria and nephrocalcinosis (FHHNC) (52, 53, 54).[Table tbl5]

**Table 5 tbl5:** Causes of low magnesium and drugs used.

Causes of low magnesium •Decreased intake •Decreased intestinal absorption (malabsorption, short bowel syndrome, severe vomitting, diarrhea, steatorrhea) •Familial hypomagnesemia with secondary hypocalcemia (TRPM6 mutation) •Autosomal dominant hypocalcemia (activating mutation in *CASR* gene) •Familial hypomagnesemia with hypercalciuria and nephrocalcinosis (FHHNC) **Drugs** •Thiazides, furosemide •Proton pump inhibitor •Antibiotics •Antimycotics (foscarnet, amphotericin B, aminoglycosides, pentamidine, rapamycin) •Anticancer drugs (cisplatin, carboplatin) •Immunosuppressants (calcineurin inhibitors-tacrolimus, cyclosporine A) •EGF-receptor inhibitors (cetuximab)

Loss-of-function mutations in TRPM6 channels affect active transcellular Mg^+2^ transportation in both the kidney and the bowel and result in hypomagnesemia with secondary hypocalcemia (HSH) (55, 56). Severe hypomagnesemia blocks PTH synthesis and secretion by increasing inhibitory G alpha subunit activity (57).

A resistance to PTH at the skeletal level is also seen in hypomagnesemia as intracellular Mg^+2^ is a cofactor of adenylate cyclase and decreases in intracellular Mg^+2^ lead to a resistance to PTH (57). Magnesium plays a key role in calcium homeostasis and should be evaluated and normalized. A more accurate assessment of magnesium stores can be obtained by measuring the red blood cell magnesium (47). In the presence of hypomagnesemia assessing urinary magnesium can be helpful. Measuring the fractional excretion of magnesium enables differentiation of renal magnesium losses from intestinal losses (FEMg = urine Mg × plasma Cr/0.7 × plasma Mg × urine creatinine × 100%). If FEMg >4% in the presence of hypomagnesemia, this would be consistent with renal magnesium wasting (58). An extra renal cause of magnesium loss is likely to be present if FEMg <2% (58).[Table tbl6]

**Table 6 tbl6:** Causes of high serum magnesium.

•Chronic kidney disease •Familial hypocalciuric hypercalcemia •Excess intake (cathartics, laxatives, parenteral Mg^+2^) •Tocolytic therapy for eclampsia

### 9. Mitochondrial disorders associated with hypoparathyroidism

Kearns–Sayer syndrome is characterized by opthalmoplegia ptosis, retinitis pigmentosa, cardiomyopathy, cardiac conduction blocks as well as ataxia.

Mitochondrial myopathy, encephalopathy, lactic acidosis, stroke-like episodes (MELAS) syndrome presents with muscle weakness and affects the central nervous system (59, 60).

### 10. Maternal hyperparathyroidism

Infants exposed *in utero* to maternal primary hyperparathyroidism may develop suppressed parathyroid function and hypocalcemia (61, 62, 63).

#### PTH resistance syndromes

Pseudohypoparathyroidism (PHP) is characterized by target organ resistance to PTH. The biochemical profile is the same as seen in hypoparathyroidism with low serum calcium and high phosphate; however, PTH levels are elevated. PHP type 1 is associated with maternal loss of function mutations in the *GNAS1* gene, which encodes the alpha subunit of the stimulatory G protein (Gsa) coupled to the PTH receptor. These mutations result in the inability of the G protein to activate adenylate cyclase upon binding of PTH to its receptor leading to failure of signal transduction to produce an end-organ response to PTH (64). The manifestations of PHP depend on whether the mutation is maternally or paternally transmitted (65) (Table 3).

#### PHP type 1a (**GNAS1 mutation on maternally inherited allele)**

The clinical features of PHP type 1a include Albright hereditary osteodystrophy (AHO) – which refers to a constellation of developmental and skeletal defects, which include short stature, obesity, dental hypoplasia, mental retardation, frontal bossing, short neck, round face, subcutaneous calcification, shortened fourth and fifth metacarpals and metatarsals (brachydactyly) and developmental delay. Resistance to other G-protein-coupled hormones may be present including gonadotropins, TSH and GHRH (66, 67). The biochemical profile demonstrates low calcium, high phosphate and high PTH.

#### PHP 1b (GNAS1 mutation or STX gene mutation)

This condition is associated with a mutation in the cis-acting element of GNAS1 resulting in reduction or complete absence of alpha subunit of stimulatory G protein in the proximal renal tubules. The PTH resistance is confined to the kidney (68, 69). There are no clinical features of AHO. The biochemical profile demonstrates PTH resistance with low calcium, high phosphate and high PTH. Usually there is no resistance to other hormones.

#### PHP1c (GNAS mutation on maternally inherited allele)

Clinical features of AHO are present as well as resistance to PTH and other hormones.

The GNAS1 mutation affects coupling of G proteins to the PTH receptor. Gsa is normal in quantity and activity, ability to stimulate adenylate cyclase is intact (70).

#### Pseudo-PHP (GNAS mutation on paternally inherited alleles)

This condition is secondary to paternally transmitted inactivating GNAS1 mutation. AHO is present without the renal tubular resistance to PTH. The paternal allele of GNAS1 is always silenced due to genetic imprinting and if the maternal allele is normal, the biochemical profile is normal (71). Laboratory profile includes normal calcium, phosphate and PTH.

#### Progressive osseous heteroplasia

A paternally inherited inactivating mutation in the *GNAS1* gene results in progressive osseous heteroplasia (72). The clinical features include extensive ectopic bone formation in the skin, muscles and connective tissues as well as AHO. Laboratory profile includes normal calcium, phosphate and PTH.

## Idiopathic

If the cause of the hypoparathyroidism is not identified following a careful clinical and laboratory evaluation, the condition can be confirmed as idiopathic in etiology.

**Key points:**

The diagnosis of hypoparathyroidism is made in the presence of low serum calcium (ionized or adjusted for albumin) and low or inappropriately normal PTH confirmed on two separate occasions. Further evaluation enables identification of the cause of hypoparathyroidism in nonsurgical cases. In young individuals or consanguineous parents a referral to a geneticist is advised for appropriate genetic counseling. DNA studies enable a molecular diagnosis to be made.

**Quality of evidence:** low.

(b) How do we evaluate target organ damage in hypoparathyroidism?

Hypercalciuria is commonly seen in chronic hypoparathyroidism on treatment, and may lead to nephrocalcinosis, nephrolithiasis and renal insufficiency. Renal complications are assessed by regular monitoring of renal function (eGFR and serum creatinine) as well as assessment for hypercalciuria with a 24-h urine calcium and creatinine assessment (5, 73, 74, 75). Renal imaging with ultrasound is recommended with identification of nephrocalcinosis or nephrolithiasis (76, 77). If ocular complications (posterior subcapsular cataracts) are suspected, a slit lamp examination is performed (5, 73, 74). The development of neurocognitive decline, movement disorders or seizures may indicate brain calcification, which are evaluated by brain imaging and an electroencephalogram. In the absence of these symptoms, the role of brain imaging to monitor these complications is unclear.

**Key points:**

The risk for and extent of renal complications are assessed by evaluating renal function (eGFR), a 24-h urine for calcium excretion, and a renal ultrasound for the presence of nephrocalcinosis or nephrolithiasis. Brain imaging is advised in the presence of cognitive impairment, movement disorders or seizures. Opthalmoscopic examination enables assessment for the development of cataracts and can be completed in the presence of symptoms or concerns.

**Quality of evidence:** low.

(c) How frequently should biochemical and clinical assessment be performed?

Current consensus supports regular monitoring of (a) serum chemistries including calcium corrected for albumin (or ionized calcium), phosphorus, magnesium, urea nitrogen and creatinine every 3–6 months; (b) urinary calcium excretion, creatinine and sodium (either by 24-h urine collection or random urine collection) and 25-hydroxyvitamin D levels (annually); (c) renal calcification by imaging, such as ultrasound, if there is persistent hypercalciuria, a history of renal stones, abnormal urinalysis or a decline in eGFR; (d) signs and symptoms of hypocalcemia and hypercalcemia should be evaluated every 6 months based on the stability of hypoparathyroidism. Asking the patient regarding their overall function is also of value as many symptoms of hypoparathyroidism are nonspecific and include low energy or fatigue or brain fog and may be a marker of poor serum calcium control and (e) basal ganglia calcification can be evaluated with brain CT or susceptibility-weighted MRI (SW-MRI) and the presence of cataracts as part of the routine annual eye examination. The serum calcium and phosphate may require closer monitoring (every 1–2 weeks) if changes are made in the dose of the calcium or active vitamin D or if symptoms are present.

**Key points:**

Serum and urine biochemistries should be evaluated at least every 6 months for calcium (ionized or albumin-adjusted), phosphate, magnesium, 25-hydroxyvitamin D and annually for 24-h urine calcium and creatinine. Following changes in the dose of calcium or active vitamin D the lab profile should be repeated in 1–2 weeks.

**Quality of evidence:** low.

## 2. Management of hypoparathyroidism – avoiding complications of treatment

(a) Are long-term complications a real problem?

Long-term complications include renal impairment, renal stones, nephrocalcinosis as well as cataracts and calcification of the basal ganglia and other regions of the brain (5). Several retrospective studies have shown that long-term complications of hypoparathyroidism are commonly seen. A large cohort study determined the rate of complications in 120 patients with permanent hypoparathyroidism with a mean follow-up of 7.4 years (75). While serum calcium levels were maintained within a calcium range of 7.5–9.5 mg/dL (1.9–2.4 mmol/L) for an average of 86% of the time, the 24-h urinary calcium analysis in 53 patients showed that 38% had a least one measurement with hypercalciuria (>300 mg/day). Renal imaging in 54 patients showed renal calcification was present in 31% of the patient population. Compared with age-appropriate historical controls from NHANES, the rates of chronic kidney disease stage 3 or higher were 2- to 17-fold greater in those with hypoparathyroidism. These data have been recently confirmed in another series of 90 patients with chronic post-surgical hypoparathyroidism (6).

In a large case–control study from a national registry in Denmark, the hazard ratio (HR) for developing kidney stones and renal insufficiency in patients with post-surgical hypoparathyroidism was 4.8 and 3.1, respectively (78). In another Danish case–control study, the HR in patients with nonsurgical hypoparathyroidism for developing renal insufficiency was 6.0 (79).

Cataracts have been reported in approximately 50% of patients with chronic hypoparathyroidism in case series (80, 81). In the case–control studies from Denmark, the risk of cataracts was increased in patients with nonsurgical hypoparathyroidism (HR 4.32) (79), but not in patients with post-surgical hypoparathyroidism (78).

Intracranial calcifications, in particular in the basal ganglia, can develop in patients with hypoparathyroidism (75, 82). The exact cause is not known, however, elevated serum phosphate levels or an elevated calcium-phosphate product are thought to be a contributing factor. The clinical significance of basal ganglia calcifications is unclear; symptoms of Parkinsonism and dystonia have also been described in some cases (82).

Serum phosphate and the calcium-phosphate product should be maintained in the normal reference range (i.e. less than 55 mg^2^/dL^2^ (4.4 mmol^2^ L^2^)) as they are thought to contribute to extraskeletal calcifications and other complications when elevated (73, 83, 84). This does however require further prospective evaluation. If serum phosphate levels are high, calcium supplements can be increased and should be given with meals as they are excellent phosphate binders. The calcium-phosphate product may not correlate with basal ganglia calcification and this requires further prospective evaluation as well as other measures including serum phosphate and the 24-h urine calcium, which may be of value in predicting the presence of extraskeletal calcification (85). The dose of the active vitamin D can also be reassessed as 1,25(OH)2 increases intestinal calcium absorption as well as phosphorus absorption. Dietary modification with a low phosphate diet (low intake of meat, eggs, cola and dairy) may be implemented as needed on an individual basis. A low-salt diet is also helpful as it lowers renal calcium losses.

**Key points:**

In hypoparathyroidism, long-term renal complications and extraskeletal calcification are commonly seen and may be reduced by lowering urine calcium excretion, serum phosphorus and the calcium-phosphate product.

**Quality of evidence: **very low.

(b) How should acute hypocalcemia be managed?

Depending on the rate of onset, biochemical severity and clinical symptoms, acute hypocalcemia may require management in hospital with intravenous calcium. Calcium gluconate is the preferred salt to be administered intravenously as it is less irritating to the veins than calcium chloride. A bolus of 1–2 g of 10% calcium gluconate (corresponding to 90–180 mg of elemental calcium) in 50 mL of 5% dextrose may be administered over 20 min followed by a continuous infusion of intravenous calcium with 1–3 mg/kg/h of elemental calcium administered as calcium gluconate. During the calcium bolus and infusion continuous cardiac monitoring is advised. Oral calcium supplements and active vitamin D are also initiated (83, 86). Hypomagnesemia should be corrected (83) and vitamin D levels should be normalized (2, 3). Low serum magnesium leads to further suppression of PTH synthesis and secretion (87). This paradoxical inhibition of the parathyroid involves intracellular signaling pathways of the CaSR with an increase in the activity of inhibitory G alpha subunits (57). Hypomagnesemia also results in a resistance to the effects of PTH in the tissues. PTH-induced bone resorption is impaired in hypomagnesemia (88, 89, 90). Intracellular Mg^2+^ is a cofactor of adenylate cyclase and decreases in intracellular ionized Mg^2+^ lead to resistance to PTH (91, 92, 93). Hypocalcemia combined with magnesium deficiency is resistant to treatment with Ca^2+^ or vitamin D, but rapidly responds to Mg^2+^ supplementation.

There are very little data (largely from case reports) regarding the possible use of rhPTH (1–84) in the management of acute hypocalcemia (94, 95, 96). Hypoparathyroidism is associated with impaired hydroxylation of 25 hydroxyvitamin D in the kidney as PTH stimulates the formation of 1,25 dihydroxyvitamin D (calcitriol) (97). Therefore, individuals with hypoparathyroidism have a deficiency of both PTH as well as calcitriol. Active vitamin D metabolites are necessary to correct the hypocalcemia and enhance the intestinal absorption of both calcium and phosphate. Calcitriol can be initiated with doses of 0.25 µg twice daily and gradually titrated upward to a dose of 2.0 µg BID if necessary (2). Occasionally higher doses may be necessary. The half-life of calcitriol is 5–8 h and the dose can be increased in 48–72 h. Alfacalcidiol (1alpha (OH) D3) can also be used in doses of 0.5–4 µg once daily; however, it has a longer time to offset of action (5–7 days) and is not as potent as calcitriol (98, 99). Close titration is required to avoid hypercalciuria and hypercalcemia which may contribute to the long-term complications of renal and extra skeletal calcification. Parent vitamin D (D2 or ergocalciferol or D3 cholecalciferol) is of value to ensure that the 25hydroxyvitamin D levels are in the normal reference range (75 nmol/L or higher) (100).

**Key recommendation:**

Acute severe hypocalcemia is treated with IV calcium boluses followed by a continuous calcium infusion as well as oral calcium supplements and active vitamin D. Hypomagnesemia must be corrected.

**Quality of evidence: **low (standard practice).

(c) What are practical strategies to lower urinary calcium?

Reducing the filtered renal load of calcium by decreasing serum calcium is the most effective method to lower urinary calcium in chronic hypoparathyroidism. Thiazide diuretics may also be effective in lowering urinary calcium losses especially when combined with a low-salt diet (101). For individuals with ADH type 1 (ADH 1) and ADH 2, thiazide diuretics may further exacerbate the hypokalemia; therefore, extreme caution must be exercised. Distributing calcium supplements evenly throughout the day can avoid peaks of serum calcium, which may contribute to hypercalciuria. Finally, rhPTH(1–84) replacement therapy may also be considered as discussed below (74).

**Key recommendation:**

Reduce urinary calcium losses with a low-salt diet and consider hydrocholorothiazide, chlorthalidone or indapamide as tolerated. In the presence of renal complications rhPTH(1–84) may also be considered.

**Quality of evidence:** low.

## 3. Management of hypoparathyroidism in pregnancy and lactation

(a) What are the physiologic changes in serum calcium and the calcium-regulating hormones during pregnancy?

During pregnancy, calcium requirements increase in order to meet the growing needs of the developing fetal skeleton (102, 103). Approximately 80% of the calcium, phosphorus and magnesium present in the full-term fetal skeleton is accreted during the 3rd trimester (102).

In pregnancy, the intravascular volume expands and with hemodilution, the serum albumin decreases resulting in a low total serum calcium (104, 105). The ionized calcium, as well as the albumin-corrected total calcium level remains unchanged in normoparathyroid mothers. PTH-related protein (PTHrP) begins to rise in the 3rd to the 13th week of gestation reaching a three-fold increase in PTHrP levels by term (106, 107, 108, 109). The source of the PTHrP is the placenta and breasts, as well as the uterus (110, 111, 112).

The lactating breast produces significant levels of PTHrP, which is released into the maternal circulation and enhances renal calcium reabsorption and phosphate excretion as well as bone resorption. PTHrP stimulates Cyp27b1 with increased production of calcitriol, which in turn enhances intestinal calcium and phosphate absorption (113, 114, 115).

Calcitriol rises in the first trimester and increases two- to three-fold by term as observed in longitudinal studies (106, 116, 117, 118, 119, 120). Longitudinal studies have confirmed a rise in both free and bound calcitriol (106, 116, 118). Vitamin D-binding proteins rise by approximately 20–40% during pregnancy and the free and bound calcitriol rises by two- to three-fold resulting in enhanced intestinal calcium and phosphate and enable the mother to achieve a positive calcium balance in the first trimester (102). The rise in calcitriol increases intestinal calcium absorption and suppresses PTH. The kidneys are the main source of the calcitriol with possibly some contribution from other tissues including the placenta and the fetus. Renal expression of Cyp27b1 is 35-fold higher in maternal kidneys than in the placenta (121). Anephric women on dialysis have low calcitriol levels during pregnancy (122). However, the factors that stimulate renal Cyp27b1 are still not clear. PTHrP, estradiol and prolactin may stimulate renal Cyp27b1 during pregnancy. PTHrP is a weak stimulator of Cyp27b1 in comparison to PTH (123). The 25(OH) D levels are stable during pregnancy despite increased conversion to calcitriol and transfer to the fetus (102, 124). Calcitriol levels fall into the pre-pregnant range during lactation and intestinal calcium absorption normalizes (120, 125, 126, 127, 128, 129). Further prospective evaluation of parent and active vitamin D supplementation is required in hypoparathyroid pregnant women (130).

PTH is suppressed to low normal or below normal levels in the first trimester and reaches mid normal levels by term (106, 108, 117, 125, 126, 131, 132). The PTH levels are impacted by changes in dietary calcium and vitamin D (102, 133). During lactation, PTH levels are suppressed or undetectable as noted with the intact PTH assays (102, 125, 133, 134, 135, 136).

Calcitonin increases during pregnancy and may protect the maternal skeleton from demineralization (102). The source of calcitonin includes the thyroid C-cells as well as the breast and placenta (137, 138). Higher levels of estradiol, estrone and estriol may result in increases in serum calcitonin (102). During lactation, calcitonin levels may be normal or increased (105, 118, 126, 139).

In pregnancy, the hypoparathyroid mother requires careful monitoring of serum calcium, phosphate and urinary calcium excretion as the physiologic rises in calcitriol and PTHrP may lower the requirements for calcium and calcitriol during pregnancy. However, case reports of hypoparathyroid women requiring higher doses of calcium and calcitriol during pregnancy have also been published (140, 141, 142, 143). Close monitoring is required during pregnancy to ensure that serum calcium is maintained in the normal reference range.

**Key finding:**

Calcium requirements increase during pregnancy due to fetal demands; however, due to rises in PTHrP and calcitriol, the dose requirements for calcium and active vitamin D in hypoparathyroid mothers may decrease. However, in some patients, dose requirements may increase.

During lactation calcitriol levels normalize. PTHrP levels are high and increase bone resorption and enhance renal calcium reabsorption. These effects may lower the dose requirements for calcitriol and calcium supplementation during lactation in hypoparathyroid patients.

**Quality of evidence: **low.

(b) What are the treatment targets for serum calcium during pregnancy?

As calcium requirements increase during pregnancy it is important to ensure that the hypoparathyroid mother is receiving adequate calcium and calcitriol supplementation and is not hypocalcemic as this can result in the development of secondary hyperparathyroidism in the fetus with fetal skeletal demineralization. In contrast, if the mother is hypercalcemic the fetal parathyroid tissue may become suppressed and the neonate may develop hypocalcemia. It is important to closely monitor patients in order to avoid hypocalcemia and hypercalcemia. Uterine irritability increases with hypocalcemia, and this may increase the risk of preterm labor (144).

It is recommended that the total calcium corrected for albumin be maintained in the low to mid normal reference range and this should be evaluated every 3 weeks during pregnancy or more frequently if changes in the calcium supplements or calcitriol dose are being made.

**Key recommendation:**

Serum calcium (corrected or ionized) should be maintained in mid-to-low normal reference range. Serum calcium, phosphorus, eGFR and magnesium should be monitored during pregnancy every 3 weeks. If changes in the dose of calcium or calcitriol are recommended, repeat the serum calcium in 1–2 weeks. Maintain the 25-hydroxyvitamin D and 24-h urine calcium in the normal reference range.

**Quality of evidence: **very low.

(c) How should management be modified to reduce the risk of maternal and fetal complications?

Close monitoring of circulating levels of calcium corrected for albumin, phosphate and magnesium is necessary to avoid complications of hypoparathyroidism in pregnancy. Uterine irritability increases with hypocalcemia and may increase risk of preterm labor or miscarriage (144). Ensure that the mother is receiving adequate calcium and calcitriol supplementation to maintain the serum calcium in the normal reference range. Neonatal complications include small for gestational age and respiratory distress syndrome (140, 145, 146, 147, 148).

A recent retrospective audit of an Australian database of ten pregnancies with hypoparathyroidism demonstrated that the requirements for calcitriol vary during the second half of pregnancy and decrease substantially during lactation (148, 149).

Hydrochlorothiazide should be avoided during pregnancy as it is classified as an FDA pregnancy risk category B drug (150). PTH has not been adequately evaluated in pregnancy. It is classified as an FDA pregnancy risk category C drug. A case of one patient treated with PTH(1–34) has been reported and the neonate was normal at birth with normal serum calcium (151). The dose of the calcitriol can be adjusted based on the serum ionized or albumin-adjusted calcium with close monitoring during pregnancy every 2–3 weeks for serum calcium (albumin-adjusted or ionized) phosphorus and magnesium. The 25-hydroxyvitamin D level should be normalized (75–125 nmol/L (30–50 ng/mL) (152).

During lactation, monitoring can continue every 4 weeks. Both pregnant and lactating women should be informed of the symptoms of hypo- and hypercalcemia (Table 7) and advised to immediately check their serum calcium if they are not well and seek urgent medical assistance as needed. Care must be co-ordinated between the treating endocrinologist as well as the obstetrician and the pediatrician to ensure optimal maternal and fetal outcomes.

**Table 7:** Symptoms of hypocalcemia and hypercalcemia.

Hypocalcemia	Numbness, tingling in face, hands and feet, muscle spasm, cramps, depression, confusion, seizures, bradyarrhythmia, wheezing, laryngospasm, congestive heart failure
Hypercalcemia	Polydipsia, polyuria, nausea, anorexia, vomiting, constipation, weakness, headaches, confusion

**Key recommendation:**

Discontinue thiazide diuretics during pregnancy, adjust the dose of calcium and active vitamin D targeting low-to-mid normal albumin-corrected calcium. Advise the pediatrician to assess the neonate immediately following birth and monitor serum calcium. During lactation continue to monitor serum calcium every 4 weeks and adjust the dose of the calcium and calcitriol supplements targeting mid-to-low normal serum calcium.

**Quality of evidence: **very low.

## 4. rhPTH(1–84) replacement therapy in hypoparathyroidism – when and how to proceed?

A number of key questions arise when considering which patients with chronic hypoparathyroidism should be treated with rhPTH(1–84) replacement therapy. Replacement therapy with rhPTH(1–84) has been approved as an adjunct to conventional therapy by regulatory agencies.

(a) Which criteria confirm that conventional therapy for chronic hypoparathyroidism has failed?

Limitations of conventional therapy with calcium, active vitamin D metabolites and vitamin D include an inability to alleviate the symptoms of hypocalcemia and to improve quality of life. In the absence of PTH, urinary calcium excretion is elevated and contributes to the long-term complications of hypoparathyroidism which include renal insufficiency, nephrocalcinosis as well as nephrolithiasis (153). Guidelines define failure of conventional treatment of chronic hypoparathyroidism as meeting certain criteria (73, 74): (1) inability to keep serum calcium in the lower half of reference range without symptoms of hypocalcemia, (2) failure to keep serum phosphate within the reference range, (3) inability to keep the calcium-phosphorus product below 55 mg^2^/dL^2^ (4.4 mmol^2^ L^2^), (4) failure to keep serum magnesium within the reference range, (5) inability to keep urinary calcium within the reference range for weight and gender and 6) failure to maintain long-term well-being and QOL.

In addition, compliance with conventional therapy is often poor and is contributed to by the large number of pills required daily as well as gastrointestinal side effects of supplemental calcium.

**Key recommendation:**

Failure of conventional therapy is confirmed in the presence of poor control of serum calcium, the presence of complications of hypoparathyroidism or the presence of a poor quality of life.

**Quality of evidence: **low-moderate.

(b) When should rhPTH(1–84) replacement therapy be considered in patients with chronic hypoparathyroidism?

PTH replacement therapy was initially evaluated in hypoparathyroidism with the PTH(1–34) molecule. Subcutaneous twice daily injections of PTH(1–34) maintained mean urine calcium in the normal range, with no difference compared to calcitriol (154, 155), whereas intravenous administration using a pump resulted in a marked decline in mean urine calcium well within the normal range, with a significant difference in comparison to calcitriol (156). More recently, PTH(1–34) in doses of 20 µg BID led to reductions in the dose of calcium and calcitriol required daily and increased serum calcium while lowering serum phosphate (157, 158) (Table 8).

**Table 8:** PTH replacement therapy – evidence table.

Study	Population		Duration of study	Study description			Effects			Quality of evidence
Age range	No. of participants	Design	Intervention	Comparator	Effect on phosphate	Effect on calcium	Effect on urinary calcium
(154)	(18–70)	27	3 years	Randomized, parallel group, open-label trial	PTH(1–34) (*n* = 14)	Oral calcitriol and calcium supplementation (*n* = 13)	No significant difference between intervention and control group (mean (s.e.) phosphorus levels were 4.6(0.08) and 4.5(0.05) mg/dL respectively)	No significant difference between intervention and control group (mean (s.e.) calcium levels were 1.92(0.02) and 2.0(0.01) mmol/L respectively)	PTH(1–34) normalized urinary calcium excretion whereas elevated levels were reported in the control group (mean (s.e.) 5.8(0.27) vs 8.2(0.51) mmol/24 h, respectively)	Moderate to high
(155)	(5–14)	12	3 years	Randomized, parallel group, open-label trial	PTH(1–34) (*n* = 7)	Oral calcitriol, calcium and cholecalciferol supplementation (*n* = 5)	No significant difference between intervention and control group (*P* = 0.13) and phosphate levels remained above the normal range throughout the 3 years. However a significant downward trend in phosphate levels was seen over time in the PTH 1–34 group (*P* < 0.01)	No significant difference between intervention and control group (mean (s.d.) calcium levels were 1.96(0.04) and 1.99(0.05) mmol/L respectively)	No significant difference between intervention and control group (*P* = 0.73)	Moderate to high
(156)	(7–20)	12	26 weeks (13 weeks each arm)	Randomized, crossover trial	(PTH) 1–34 injection and cholecalciferol	(PTH) 1–34 delivered by an insulin pump and cholecalciferol	Pump delivery of PTH1–34 had higher phosphate levels (*P* < 0.01)	Pump delivery of PTH1–34 produced near normalization of serum calcium (*P* < 0.02)	No significant difference between pump delivery of PTH1–34 and injection (*P* = 0.3). However, pump delivery normalized the urinary calcium	Moderate to high
(159)	(31–78)	62	24 weeks	Double-blind, placebo-controlled, randomized trial	PTH(1–84) adjunct to vitamin D analogs and calcium supplementation (*n* = 32)	Placebo and vitamin D analogs and calcium supplementation (*n* = 30)	PTH1–84 decreased phosphate levels (*P* < 0.05)	PTH1–84 increased ionized calcium levels (*P* < 0.01)	PTH1–84 increased urinary calcium excretion (*P* < 0.01)	High
(160)	(18–85)	134	24 weeks	Double-blind, placebo-controlled, randomized trial	PTH(1–84) adjunct to vitamin D analogs and calcium supplementation (*n* = 90)	Placebo and vitamin D analogs and calcium supplementation (*n* = 44)	PTH1–84 decreased phosphate levels (*P* = 0.0025)	PTH1–84 increased albumin-corrected serum calcium concentrations at the beginning of treatment then remained relatively stable	No significant difference in the change of urinary calcium levels between intervention and control group (*P* = 0.57)	High
(161)	(26–72)	33	6 years	Open-label trial	PTH(1–84)	n/a	No significant change in phosphate levels at 6 years. However, a decrease in phosphate levels was observed at years 4 and 5 (*P* = 0.01)	No significant change in calcium levels at the end of study period (*P* = 0.53)	PTH1–84 significantly decreased urinary calcium levels at 6 years (*P* = 0.007)	Low

Replacement therapy with PTH(1–84) maintains serum calcium and phosphate levels in the appropriate range, while reducing the daily doses of calcium and active vitamin D metabolites (159, 160). In some patients PTH replacement therapy enables withdrawal of calcium and active vitamin D analogs. The effects on urinary calcium excretion are modest; however, a long-term open-label study suggests a progressive decrease in urinary calcium excretion (161) (Table 8). Skeletal abnormalities (low turnover status, increased bone mineral density (BMD)) are improved. Bone turnover markers increase within 1 year and subsequently decline to levels that are higher than pretreatment values. BMD increases at the lumbar spine and to a lesser extent at the hip, while there is a progressive decline at the distal 1/3 radius (161). Bone histomorphometry studies have shown reductions in trabecular width and an increase in trabecular number. Intratrabecular tunneling has been demonstrated in about half of the biopsy specimens (161). Cancellous bone matrix mineralization is markedly increased in hypoparathyroid bone compared to normal. rhPTH(1–84) treatment after 1 year is associated with a decrease in the degree of mineralization which returns to the baseline value at year 2 (162). Conversely, the greater heterogeneity detected at 1 year persists (162).

Recently, an increased rate of vertebral fractures has been reported in patients with idiopathic hypoparathyroidism treated with conventional therapy (81). Replacement therapy with rhPTH(1–84) may have positive skeletal effects on bone strength and fracture risk; however, this requires further evaluation (Table 9).

**Table 9:** GRADE evidence profile of RCT for rhPTH(1–84) therapy.

Certainty assessment							Impact	Certainty	Importance
No. of studies	Study design	Risk of bias	Inconsistency	Indirectness	Imprecision	Other considerations
Urinary calcium levels									
2	RT	Not serious	Not serious	Not serious	Very serious^a^	None	No significant differences between mean urinary calcium excretion in intervention and control group	⊕⊕◯◯ low	Critical
Phosphate levels									
2	RT	Not serious	Not serious	Not serious	Very serious^a^	None	Serum phosphate levels decreased significantly in the intervention group relative to the control group	⊕⊕◯◯ low	Critical
Calcium levels									
2	RT	Not serious	Not serious	Not serious	Very serious^a^	None	Albumin-corrected total calcium levels were stabilized while elevated ionized calcium levels were observed	⊕⊕◯◯ low	Critical

*Question:* Effects of RhPTH1–84 compared to conventional therapy on serum phosphate, urinary calcium and serum calcium for adult hypoparathyroidism.

Adapted from Sikjaer *et al*. (159), Mannstadt *et al*. (160).

RT, randomised trials; ^a^ few number of participants.

Several studies have shown that quality of life is reduced in patients with hypoparathyroidism (163, 164, 165, 166). Short-term placebo-controlled studies have shown either no effect or modest improvement, whereas a long-term open-label study has shown a benefit in all parameters of the SF-36 scale (163).

PTH replacement therapy is well tolerated and adverse events are mild and transient.

No data are currently available on the potential long-term benefits of rhPTH(1–84) replacement therapy. The FDA has approved rhPTH(1–84) with a ‘black box’ warning because of an increased risk of osteosarcoma in rats treated with high doses of PTH(1–34); however, an increased rate of osteosarcoma has not been observed in humans despite use in more than a million people (167).

As PTH therapy in hypoparathyroidism has been demonstrated to lower the requirements for calcium and active vitamin D analogs and also lower serum phosphate as well in some studies demonstrated reductions urinary calcium excretion, it has been proposed that PTH replacement be considered in the following circumstances;

1. inadequate control of serum calcium,
2. oral calcium or vitamin D medications required to control serum calcium or symptoms that exceed 2.5 g calcium or >1.5 μg calcitriol per day,
3. hypercalciuria, renal stones, nephrocalcinosis, stone risk or reduced creatinine clearance or eGFR (<60 mL/min),
4. hyperphosphatemia and/or calcium-phosphate product that exceeds 55 mg^2^ dL^2^ (4.4 mmol^2^ L^2^) (74).

There are many factors contributing to urine calcium including the filtered calcium load as well as the dose of PTH and frequency of administration of this molecule. Further prospective data will enable refinement of administration with a goal to consistently reduce urine calcium excretion.

PTH replacement may also be of value in individuals who have malabsorption or are intolerant of large doses of oral calcium supplements as well as those who are noncompliant with taking several pills each day. PTH replacement therapy may improve quality of life; however, effects of PTH replacement on quality of life require further study as currently controlled studies have not demonstrated reversal of muscle weakness and fatigue with therapy. Wide fluctuations in serum calcium may occur in certain individuals with hypoparathyroidism particularly following exercise or with intercurrent illness and may result in hospitalization. Overcorrection of hypocalcemia may lead to hypercalcemia and individuals with wide fluctuations in serum calcium require close monitoring ideally with a calcimeter which can provide immediate measures of serum calcium in real time. Having such devices easily available to patients will enable assessment of serum calcium with the onset of symptoms and allow closer titration of therapy based on current serum calcium. Such close monitoring is expected to revolutionize care for hypoparathyroidism similar to the enhanced care possible for diabetes with the advent of glucometers. Calcimeters have been developed and are expected to be released for general use in the near future. The approved indications for PTH replacement therapy may vary depending on the regulatory authorities of each country.

**Key recommendation:**

rhPTH(1–84) replacement therapy may be considered if the serum calcium is poorly controlled, high doses of calcium or active vitamin D are required, renal complications are present or quality of life is poor or gastrointestinal malabsorption is present.

**Quality of evidence: **low.

(c) Once rhPTH(1–84) replacement therapy is started, how should therapy be titrated?

The FDA approved starting rhPTH(1–84) at a dose of 50 µg by subcutaneous injection once each day with a concomitant 50% decrease in the dose of active vitamin D metabolites (83, 167). After an appropriate interval of several weeks, the dose may be increased to 75 µg each day, and then 100 µg each day after several more weeks, or reduced to 25 µg each day, as required to meet treatment goals, aiming to discontinue the active vitamin D and reduce calcium supplements to 500 mg daily.

**Key recommendation:**

rhPTH(1–84) replacement therapy can be initiated at a 50 µg daily dose with close monitoring of serum calcium and phosphate. The dose of rhPTH (1–84) can be gradually titrated upwards or downwards based on the lab profile and the doses of serum calcium and active vitamin D can also be gradually reduced as the dose of the rhPTH(1–84) is gradually increased.

**Quality of evidence: **low-moderate.

(d) If rhPTH(1–84) replacement therapy is initiated, should it ever be stopped?

This is left to the judgment of the treating physician. rhPTH(1–84) replacement therapy may be stopped if treatment goals cannot be achieved despite appropriate dose adjustment, or patients are unable to tolerate or comply with therapy for any reason. Discontinuation of rhPTH(1–84) requires gradual reductions in the dose over several weeks as abrupt cessation has been associated with hypocalcemia, which may reflect increased bone remodeling favoring formation leading to the hungry bone syndrome (168). While decreasing the rhPTH(1–84) dose treatment with active vitamin D metabolites should be restarted or adjusted, as appropriate.

**Key recommendation:**

Stop therapy if the treatment goals cannot be achieved despite appropriate dose adjustment or if the patient is unable to tolerate or comply with therapy for any reason.

**Quality of evidence: **low-moderate.

## Conclusion

Hypoparathyroidism is a rare condition, which requires careful evaluation and timely pharmacologic intervention in order to prevent significant morbidity and mortality. Frequent laboratory monitoring of the biochemical profile and patient education is essential to achieving optimal control of serum calcium and potentially lowering the risk of long-term complications.

Pregnancy requires close monitoring to ensure that the mother maintains a serum calcium in the low normal reference range with avoidance of hypercalcemia as well as hypocalcemia for optimal maternal and fetal outcomes.

Conventional therapy consists of calcium and active vitamin D. PTH replacement therapy has been demonstrated to lower the doses of calcium and active vitamin D required and may lower the long-term complications of hypoparathyroidism.

## Declaration of interest

A K: Research funds from Shire; C K: Novartis Pharma, Advisory Board Consultant, Shire Pharmaceuticals, Principal Investigator, A European Post-Authorisation Observational Study (Registry) of Patients With Chronic Adrenal Insufficiency (AI), Springer Publisher, Royalty Honoraria (Book, Journals), Elsevier Publisher, Royalty Honorarium (Journal); S V U: Relationships with for-profit and not-for-profit interests: Grants/Research support: Novartis, Sanofi, Speakers Bureau/Honoraria: Abbott, Acerus pharmaceuticals, Novartis, Ipsen, Sanofi, Consulting Fees: Pfizer; J-P B: No conflict of interest to declare; J B: No disclosures; M L B: Received honoraria from Amgen, Bruno Farmaceutici, Kyowa Kirin; Academic grants and/or speaker: Abiogen, Alexion, Amgen, Bruno Farmaceutici, Eli Lilly, Kyowa Kirin, MSD, NPS, Servier, Shire, SPA; Consultant: Alexion, Bruno Farmaceutici, Kyowa Kirin, Servier, Shirel; C M: Consultant and speaker for Abiogen-Pharma and SHIRE, Research grant from SHIRE; L R: Speakers fee: Shire, Alexion, Eli Lilly, Amgen, Takeda Pharmaceuticals; Consultancy: Shire, Alexion and Kyowa Kirin; R R: Advisory board or speaker for Radius Health, Sandoz, Effryx and Theramex; M Z S: Grants/Research Support: Eli Lilly, Valeant, Novo Nordisk Speakers Bureau/Honoraria/Consulting Fees: Novo Nordisk, Amgen, Merck; B Y: Nothing to disclose; R T: Research income from: Medical Research Council programme grant, Wellcome Trust Investigator Award, NIHR Senior Investigator, NIHR Translational Research Collaboration, NIHR Oxford Grant – BRC funding, Wellcome Trust clinical training fellowships, EU ITN Marie Curie grant, Glaxo-SmithKline research grant, Kidney Research UK (KRUK) project grant, Novartis Research Grant, NPS Pharmaceuticals (USA), Marshall Smith Syndrome Research Fund. Chairman of Astra-Zeneca Stratified Medicine Panel, Honoraria/lecture and consultancy fees from Novartis, Lilly, AstraZeneca and Ipsen; B C: Research grant support and consultant for Shire, Inc., Data monitoring board member for Amgen, Inc., Data monitoring board member for GSK.

## Funding

Funding was received from Canadian Endocrine Update, McMaster University and Western University for the completion of the literature review – 2018.
